# Simultaneous fermentation and enzymatic biocatalysis—a useful process option?

**DOI:** 10.1186/s13068-024-02519-z

**Published:** 2024-05-25

**Authors:** Katharina Oehlenschläger, Emily Schepp, Judith Stiefelmaier, Dirk Holtmann, Roland Ulber

**Affiliations:** 1 Institute of Bioprocess Engineering, University of Kaiserslautern-Landau, Gottlieb-Daimler-Straße 49, 67663 Kaiserslautern, Germany; 2 Institute of Process Engineering in Life Sciences, Karlsruhe Institute of Technology, Kaiserstraße 12, 76131 Karlsruhe, Germany

**Keywords:** Simultaneous saccharification and fermentation, Enzymatic biocatalysis, Enzymatic esterification, Butyl butyrate, Biofuel, Coupling fermentation and enzymatic biocatalysis

## Abstract

Biotransformation with enzymes and de novo syntheses with whole-cell biocatalysts each have specific advantages. These can be combined to achieve processes with optimal performance. A recent approach is to perform bioconversion processes and enzymatic catalysis simultaneously in one-pot. This is a well-established process in the biorefinery, where starchy or cellulosic material is degraded enzymatically and simultaneously used as substrate for microbial cultivations. This procedure leads to a number of advantages like saving in time but also in the needed equipment (e.g., reaction vessels). In addition, the inhibition or side-reaction of high sugar concentrations can be overcome by combining the processes. These benefits of coupling microbial conversion and enzymatic biotransformation can also be transferred to other processes for example in the sector of biofuel production or in the food industry. However, finding a compromise between the different requirements of the two processes is challenging in some cases. This article summarises the latest developments and process variations.

**Graphical Abstract:**

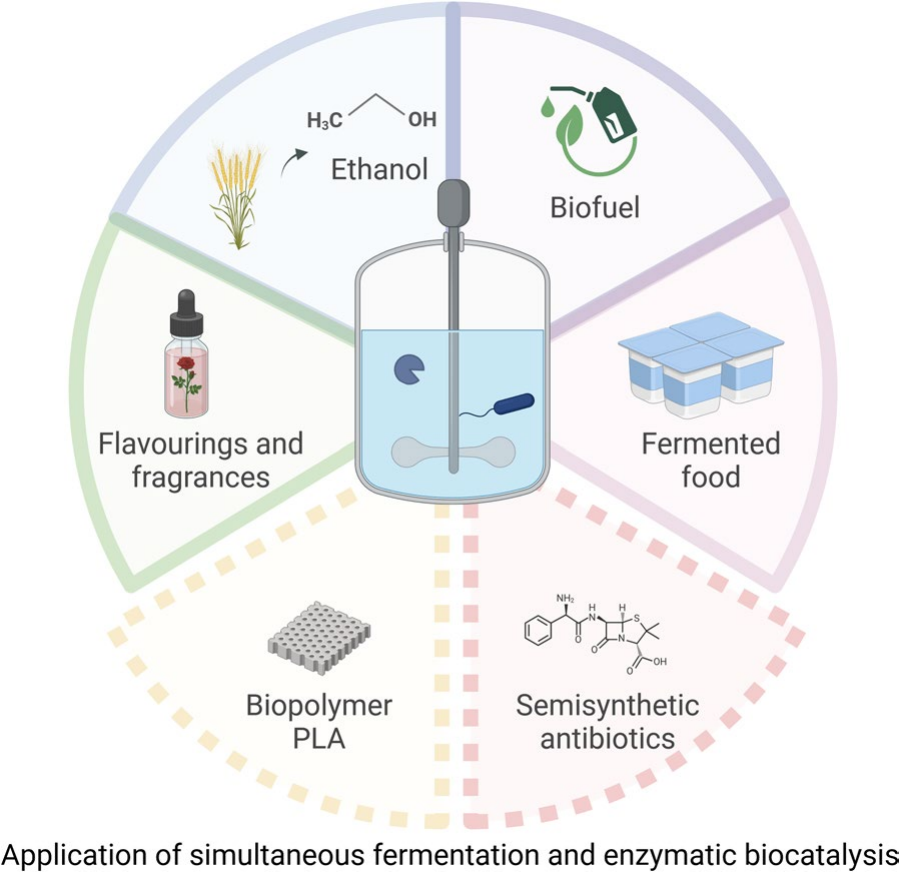

## Background

**Fig. 1:**
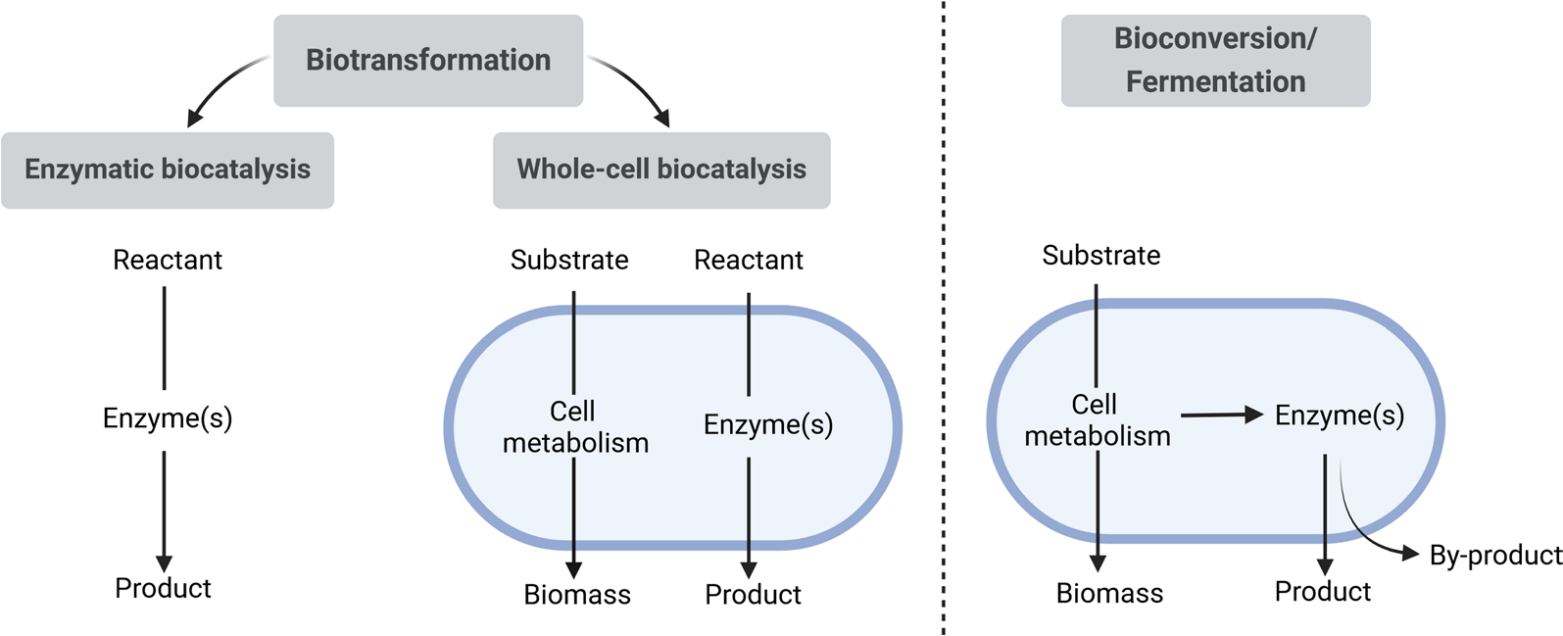
Differentiation of the terms biotransformation and bioconversion. Definition adapted from Wierckx [80]

The production of valuable products based on renewable resources is a highly relevant topic due to rising costs of mineral oil and the projected depletion of fossil resources. Switching to the use of renewable raw materials as an alternative to petroleum-based production processes is an important step towards a more sustainable industry [56]. Bioconversion is an interesting option to utilise these renewable materials as it offers a wide product range depending on the biocatalyst. The following biotransformation and bioconversion will be defined adapted from Wierckx schematically shown in Fig. 1 [80].

**Fig. 2:**
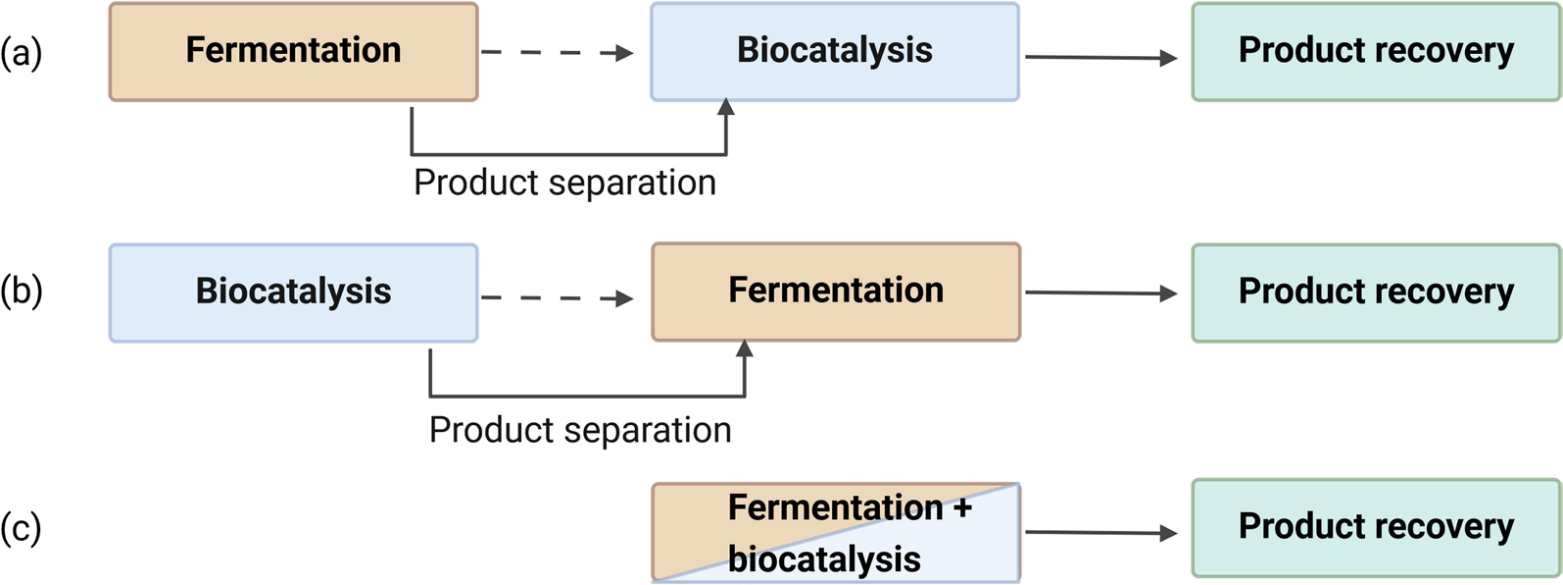
Schematic representation of process variants for the combination of fermentation and enzymatic biocatalysis. Processes shown by dotted lines exclude downstream processes and continuous lines include downstream processes between the individual process steps

According to that, biotransformation will be defined as the conversion of a reactant into products independent to cell metabolism. Whole-cell biocatalysts or enzymatic biocatalysts can be used as biocatalysts. In comparison, bioconversion describes the formation of biomass and product through the metabolism of renewable substrates. Therefore, bioconversion is coupled to the cell metabolism and can be performed by cells as biocatalysts. In the following, fermentation is defined as a bioconversion and will be used for all microbial cultivations, including alcohol fermentations. In enzymatic processes, on the other hand, the production of the biocatalyst and the catalytic reaction are separated [66]. This can, therefore, include isolated enzymes, immobilised enzymes, or whole-cells that catalyse the transformation of added molecules and are isolated from a previous fermentation. The disadvantage of using whole cells is that, in addition to the desired reaction, other metabolic reactions may take place, which can reduce the yield and lead to unwanted by-products. Moreover, cellular reactions could lead to degradation of substrate or product of the desired reaction and result in a reduction in product yield [66]. However, bioconversion and whole-cell biocatalysis naturally involve complex metabolic pathways, which include enzymatic cascades that lead from a substrate of minor value to one or more valuable products. In contrast, most enzymes only catalyse specific reactions. Therefore, coupling fermentation with enzymatic biocatalysis can be a useful approach to synthesise more complex and higher value products. A well-known example for such processes is the production of pharmaceuticals such as semi-synthetic antibiotics. Here, a fermentation is linked with a subsequent enzymatic conversion (Fig. 2a) [18]. In addition, the combination of a biocatalysis in the first step with a subsequent fermentation can be useful (Fig. 2b). For example, biorefinery utilises lignocellulosic biomass, which is first degraded enzymatically and thus made accessible for microbial fermentation [70]. A current approach is to perform the processes simultaneously in one reaction vessel (Fig. 2c).

This would simplify the process procedure, because purification steps between the fermentation and the enzymatic catalysis would no longer be necessary. In addition, the operation costs could be reduced by the reduced process duration and the elimination of one vessel. However, it is unclear to what extent the processes influence each other and whether a simultaneous approach is possible. Therefore, different processes with one-pot application are reviewed here and compared to the separated processes.

## Simultaneous saccharification and fermentation as an established process in biorefineries

Biorefinery is based on the utilisation of whole plant or complex biomass to produce a spectrum of valuable products and thus opens an alternative to traditional refinery based on crude oil [33]. To make the complex substrates (e.g., maize, lignocellulosic biomass) microbiologically usable, a pretreatment is necessary. The enzymatic treatment makes the utilisation of a wide range of substrates possible. Every starchy or cellulosic raw material can serve as a substrate for the saccharification and fermentation process. As starchy materials grains, cassava, sweet potato, sweet sorghum, corn, wheat, rice, potatoes and sugar beets are used [34]. So-called “second generation raw materials” are based on lignocellulosic compounds that do not compete with food supplies [62]. These include agricultural residues such as straw, but also green waste [59, 78]. Depending on the raw material, different levels of enzymatic pretreatment are required. For starchy materials, the enzyme α-amylase cleaves the α-D-1,4 glycosidic bond to hydrolyse starch into shorter oligosaccharides [48]. The enzyme glucoamylase catalyses the degradation from starch to glucose [35]. Moreover, the combination of several enzymes including α-amylases, glucoamylases, pullulanases, isoamylases and maltigenic amylases is usual for starch degradation [19]. The enzymatic degradation of lignocellulosic raw material requires a more extensive preparation because of the complex matrix of cellulose, hemicellulose and lignin in the wall of plant cells. Several structural components can affect the rate of hydrolysis. These are the lignin content, the crystallinity of cellulose and the accessibility of the cellulose surface for the enzymes [23]. For this reason, mechanical or chemical methods including the organosolv or hydrothermal pretreatment are used, that break up the lignocellulosic structure [76]. This pretreatment prepares the cellulose for enzymatic degradation by cellulases, which can be classified by their catalytic mode of action into endoglucanases, exoglucanases and β-glucosidases [29].

The general limitation of the enzymatic degradation of cellulose and starch is the end-product inhibition of sugars, which is present for cellulases and amylases [27, 60]. An efficient method to overcome this limitation is to couple the enzymatic hydrolysis with a microbial fermentation. The continuous sugar consumption through microorganism prevents sugar accumulation and, therefore, the inhibition of the enzymatic hydrolysis. The simultaneous process also has some further advantages like saving in time and in the number of needed vessels. Comparing the separated hydrolysis and fermentation (SHF) and simultaneous saccharification and fermentation (SSF) is, therefore, part of current research. Whether or not the coupled process can compete with the separate process in terms of productivity will be discussed in the following.

### Comparison of SSF and SHF

The best-known process for simultaneous saccharification and fermentation is the production of ethanol [64]. The commonly utilized strain for ethanol production is *Saccharomyces cerevisiae*, as it combines a good tolerance regarding temperature, pH, ethanol and other compounds present in the hydrolysate [2, 51]. This is important, because the simultaneous process requires a compromise between the optimal conditions for enzymatic hydrolysis and microbial fermentation. In addition, fermentation metabolites could negatively influence the enzymatic hydrolysis in the combined process. Therefore, a comparison between SHF and SSF is useful to assess the impact of these factors on the combined process. Table 1 summarises literature that directly compares separate and simultaneous fermentation processes with *S. cerevisiae*.

**Table 1:** Comparison of Separated Hydrolysis and Fermentation (SHF) and Simultaneous Saccharification and Fermentation (SSF) processes

**Literature**	**Substrate**	**Pretreatment**	**Process**	**Conditions**	**Ethanol concentration/g L**^**−1**^	**Percentage of theor. yield**_**EtOH**_	**Productivity/g L**^**−1**^** h**^−1^*
[16]	Empty fruit bunch	10% NaOH, 150 °C, 30 min, 4 bar	SHF	hydrolysis: 50 °C, 150 rpm, pH 4.8 fermentation: 32 °C, 150 rpm	37.4	76.0	0.52
			SSF	32 °C, 150 rpm, pH 4.8	47.7	97.0	1.98
[1]	Wheat straw	Steam explosion; 200 °C, 3–10 min; 3 × 30 min washing, NaOH solution at 65 °C	SHF	hydrolysis: 45 °C, 250 rpm, pH 4.8, fermentation: 37 °C, 220 rpm, pH 4.8	32.1	81.0	0.3
			SSF	37 °C, 220 rpm, pH 4.8	25.1	68.0	0.83
[57]	Corn stover	Wet explosion; 175 °C, 20 min; 1% NaOH, 95–100 °C, 5 h	SHF	hydrolysis: 50 °C, pH 5, 150 rpm, fermentation: 33 °C, pH 5, 150 rpm	26.8	65.3	0.16
			SSF	33 °C, pH 5, 150 rpm	28.4	69.2	0.3
[57]	Loblolly pine	Wet explosion; 175 °C, 24 min; 1% NaOH, 95–100 °C, 5 h	SHF	hydrolysis: 50 °C, 150 rpm, pH 5, fermentation: 33 °C, 150 rpm, pH	23.3	58.4	0.14
			SSF	3 °C, 150 rpm, pH 5	25.0	62.5	0.26
[83]	Cassava pulp	none	SHF	hydrolysis: 50 °C, 200 rpm, pH 5, fermentation: 37 °C, 200 rpm	23.5	43.1	0.14
			SSF	37 °C, 200 rpm, pH 5	34.7	63.6	0.29

^*^Productivity regarding the whole process, enzymatic hydrolysis and fermentation

Several authors detected a better ethanol production within the SSF process. Dahnum et al. and Zhu et al. measured an increase in ethanol concentration of 27.5% and 47.7%, respectively, in the SSF compared to the SHF process when using empty fruit bunch and cassava pulp as substrate [16, 83]. In fact, Zhu et al. were able to achieve an ethanol concentration of 34.7 g L^−1^ in the SSF process without any prior pre-treatment [83]. Compared to the separated process, the percentage of theoretical yield of ethanol was also increased by 20.5%. This increase in ethanol production within the SSF process was achieved although using the optimal process conditions for the separate steps in the SHF approach. Similarly, Rana et al. found a slight increase in ethanol concentration by 6.0% and 7.3%, respectively, when fermenting corn stover and loblolly pine [57]. The explanation for the higher ethanol production could be the prevention of the enzyme inhibition through high sugar concentrations in the SSF process, which leads to higher availability of fermentable sugars [57]. Alfani et al. describe a decrease in ethanol concentration of 21.8%, when comparing the SHF and SSF process concerning lignocellulosic material from wheat straw, which has higher hemicellulosic content [1]. A successful simultaneous fermentation, therefore, depends on the starting material and on the pre- and enzymatic treatment as well. Nevertheless, the productivity of ethanol is increased by at least a factor of 1.9 in all cases through the time-saving simultaneous process. In the direct comparison of the two process options, the simultaneous process variant appears to have a positive effect on product formation in most cases.

Using a variety of microorganisms, it is also possible to produce a range of other compounds. The butyrate producing *Clostridium thermobutyricum* is a suitable bacterium for SSF processes, as it can be cultivated at temperatures up to 50 °C [79]. This temperature tolerance makes it well-suited for SSF, as it goes along with the optimal temperature for enzymatic hydrolysis. However, the necessary pH regulation at pH 6 for the acid production is not optimal for the enzymatic step, which is why a pH shift is necessary [79]. The simultaneous fermentation of *C. thermobutyricum* yielded a butyrate concentration of 44 g L^−1^ when fermenting sugars from sweet sorghum juice [79]. The thermophilic bacterium *Bacillus smithii* is another promising candidate for the SSF process, because it can be cultivated at 55 °C. The cultivation based on the cellulosic fraction from municipal solid waste produced a lactic acid concentration of 5.1 g L^−1^ [11]. In addition, cultivation with *Aspergillus niger* for citric acid production was carried out simultaneously with enzymatic hydrolysis. This simultaneous process resulted in a final citric acid titer of 120.0 g L^−1^, which was 20.0 g L^−1^ more than in a separate process [28]. However, in this case, an additional solid/liquid separation of the hydrolysate slurry was performed during the SHF process, which resulted in sugar loss. Direct comparison with the SSF process is, therefore, difficult.

In summary, the simultaneous enzymatic saccharification and microbial fermentation approaches show promising results. The compromised process conditions used during the simultaneous variant do not appear to have a negative effect on the enzymatic conversion. Instead of this, the continuous conversion of sugar through microorganisms results in a reduction of enzyme inhibition. This led to higher product concentrations in most of the cases which were compared here (Table 1). The lower operating time also results in a higher productivity and leads to lower power consumption. In this regard, Hou and Bao calculated a 20% reduction in process costs by working with the SSF instead of an SHF [28]. Wingren et al. also calculate a reduction in process costs of nearly 10%, mainly caused by the reduction in capital costs [81]. In addition to the selection of literature presented here to compare the two process variants SSF and SHF, there are many other recent studies that address the topic of simultaneous saccharification and fermentation. These deal, for example, with the use of alternative substrates (e.g., paper sludge waste, melon peel waste) or the modelling and optimisation of the process [10, 61, 75]. Other process variants such as fed-batch fermentation are also being investigated [3]. This shows that simultaneous fermentation and enzymatic catalysis is an established method in the field of biorefinery and illustrates its potential.

## Exploring further applications for simultaneous processes

### Coupling fermentation and enzymatic esterification to produce esters

The advantages of coupled fermentation and enzymatic biocatalysis in the examples mentioned above lead to the idea to transfer it to further processes. The simultaneous fermentation and enzymatic esterification is a method for the production of ester, which can be used as flavors and represent about a quarter of the world market for food additives [41]. For this application, a product of natural origin is preferred to a chemical production method [8]. Furthermore, several esters show promising results in terms of their physical and ignition properties, making them suitable as fuel or fuel additive [13]. Therefore, a biotechnological process to produce esters offers the possibility of synthesising alternative biofuels.

**Fig. 3:**
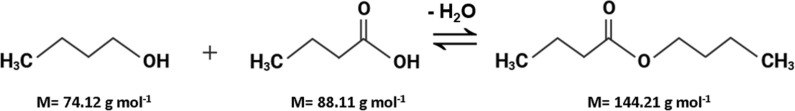
Enzymatic catalysis of butyl-butyrate synthesis using lipase

The biotechnological production of esters is possible by combining the microbial fermentation to produce an alcohol and carboxylic acid with an enzymatic esterification step. For the fermentation process, the cultivation of clostridia is sensible because of its product spectrum. Clostridia have a two-phased metabolism, consisting of the acidogenesis to produce acids and the solventogenesis for the production of solvents [47]. Nevertheless, the acid is usually only produced in low concentrations and partly reassimilated during the solventogenesis which is why additional acid must be supplemented when using solventogenic clostridia. For the esterification step, the enzyme lipase can be used. This enzyme catalyses the equimolar reaction of carboxylic acid and alcohol to the ester. The enzymatic esterification is exemplarily shown by the reaction of butanol and butyric acid in Fig. 3.

**Fig. 4:**
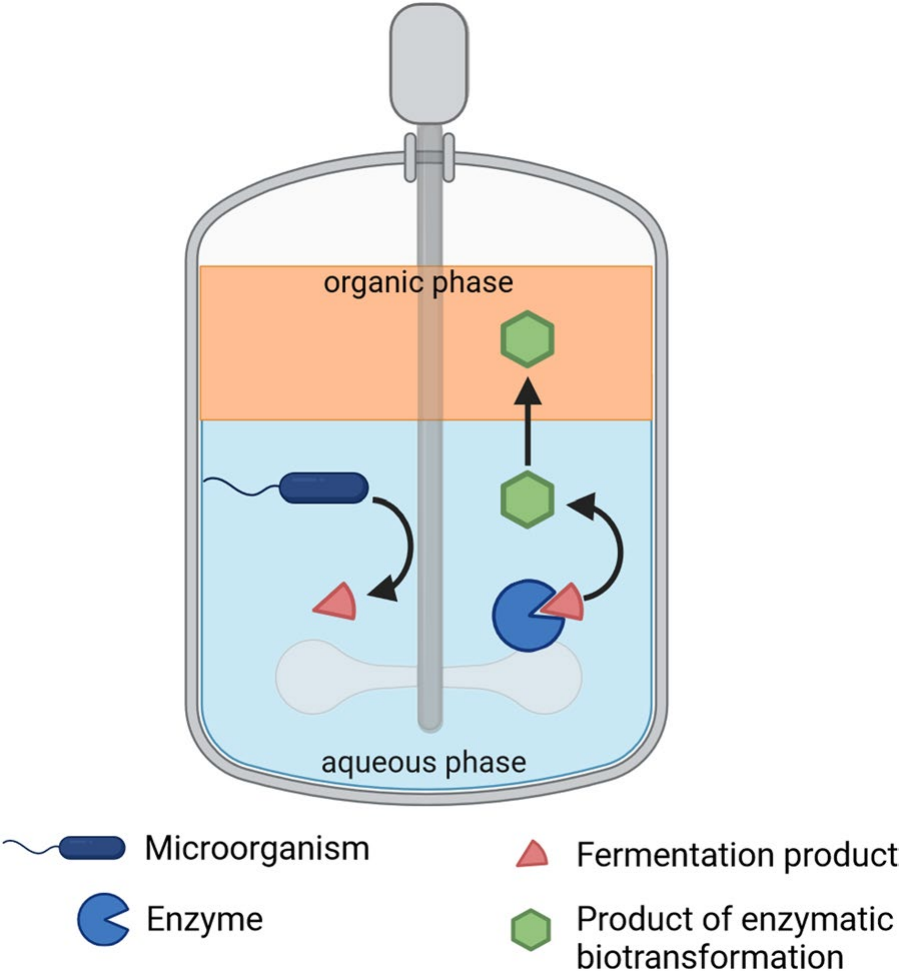
Schematic illustration of the simultaneous fermentation and esterification. Fermentation products serve as substrate for the enzymatic biotransformation. An organic phase is used for in-situ extraction of the product

The fermentation and enzymatic esterification can be combined in one-pot and, therefore, be performed simultaneously by adding lipase to the fermentation broth. As the produced ester is non-soluble in water, an organic phase must be added to facilitate the enzymatic esterification. Figure 4 shows a schematic illustration of the one-pot process to produce esters by coupling fermentation and enzymatic biotransformation.

The in-situ extraction is also an efficient downstream method to separate the ester from the fermentation broth. The product can then be separated from the organic phase by distillation. Furthermore, the reaction equilibrium is shifted to the product side by the continuous extraction of the ester into the organic phase. The choice of the extractant mainly depends on the cost, toxicity, and the partitioning coefficient describes the distribution of the component in the two phases [55]. Regarding the fermentation process the organic phase must also be biocompatible with the microorganism. Table 2 summarises literature concerning the application of coupling fermentation, esterification and in-situ extraction in a single vessel.

**Table 2:** Literature overview regarding the simultaneous fermentation and esterification

Final ester concentration/g L_aqu._^−1^	Microorganism, product	Supplemented reactants/g L_aqu._^−1^	Lipase*	Solvent ratio/V_aqu._:V_org_	Literature
Butyl-butyrate, 0.8	*C.* *acetobutylicum*, butanol, butyric acid	Butyric acid, 3.4	*Candida antarctica* lipase B, immobilised, 1.7 U mL^−1^, 2.2 g L^−1^	Hexadecane 6:1	[74]
Butyl-butyrate, 17.3	C. *tyrobutyricum,* butyric acid	Butanol continuous feed, 10	*Candida* sp. (recombinant, expressed in *A. niger*), 25 U mL^−1^, 5 g L^−1^	Hexadecane 2:1	[82]
Butyl-butyrate, 11.0	C. *beijerinckii*, butanol	Sodium butyrate, 50	*Candida* sp. (recombinant, expressed in *A. niger*), 25 U mL^−1^, 5 g L^−1^	Hexadecane 2:1	[14]
Butyl-butyrate, 5.1	*C. beijerinckii,* butanol*, C. tyrobutyricum,* butyric acid	*-*	*Candida* sp. (recombinant, expressed in *A niger)*, 25 U mL^−1^, 5 g L^−1^	Hexadecane 2:1	
Butyl-butyrate, 7.2	engineered *E. coli*, butanol and butyric acid	-	*Candida* sp. (recombinant, expressed in *A. niger*), 25 U mL^−1^, 6.7 g L^−1^	Hexadecane 1:1	[69]
Butyl-butyrate, 6.1	*C. acetobutylicum*, butanol	Butyric acid, 5	Surface display of lipase on *E coli*	Dodecane 2:1	[43]
Butyl-butyrate, 8.5	*C. tyrobutyricum,* butyric acid	Butanol, 10	Surface display of lipase on *E coli*	Dodecane 2:1	
Butyl-butyrate, 6.7	*C. acetobutylicum*, butanol, *C. tyrobutyricum,* butyric acid	-	Surface display of lipase on *E coli*	Dodecane 2:1	
Butyl-acetate, 3.6	*C*.* acetobutylicum*, butanol	Acetic acid, 15	*C. antarctica* lipase B, 100 U mL^−1^, 19.5 g L	Dodecane 2:1	[44]
Butyl-acetate, 2.9	*A*.* succinogenes*, acetic acid	Butanol, 20	*C. antarctica* lipase B, 100 U mL^−1^, 20 g L	Dodecane 2:1	
Butyl-acetate, 2.2	*A*. *succinogenes*, acetic acid, *C*. *acetobutylicum*, butanol	-	*C. antarctica* lipase B, 100 U mL^−1^, 20 g L	Dodecane 2:1	

^*^Enzyme activity calculated from manufacturer information, utilised enzyme activity and concentration related to the aqueous phase

aqu.: aqueous phase

org.: organic phase

Van den Berg et al. cultivated *Clostridium acetobutylicum* for butyrate and butanol production and supplemented lipase for the esterification to build butyl-butyrate [74]. One main advantage of this approach is to overcome the possible toxicity of the product butanol by esterification. The production of butanol is the main limitation of clostridial fermentations as a product concentration of 14 g L^−1^ stops the metabolism due to damage to the membrane functionality [52]. With the esterification, butanol is consumed and converted into the less toxic ester which enables the continuous fermentation of clostridia and, therefore, higher product concentrations. Van den Berg et al. used hexadecane for the in-situ extraction of the ester. Hexadecane can also be used as a fuel additive in diesel engines, even improving combustion and emission characteristics compared to diesel fuel [7]. In this case, the ester enriched hexadecane could be used as a fuel additive. However, considering the cost of hexadecane the recycling of the solvent would be more meaningful. The authors measured a final ester concentration of 4.9 g L^−1^ in the hexadecane phase [74]. Since the phase ratio of 6:1 (V_aqu._:V_org._) results in a heightened concentration of the ester within the organic phase, it is more logical to evaluate the product relative to the aqueous phase. In this case, the outcome is an ester concentration of 0.8 g L^−1^ which is comparatively low considering that the initial concentrations of the reactants, butyric acid and butanol were 3.4 and 5.3 g L^−1^, respectively. The esterification equilibrium can be positively affected by supplying higher educt concentrations. Zhang et al. continuously supplemented butanol in a concentration of 10 g L^−1^ [82]. Moreover, *Clostridium tyrobutyricum* was utilised for the fermentation to produce higher butyrate concentrations. With the supplementation of lipase, Zhang et al. produced an ester concentration of 17.3 g L^−1^ relating to the aqueous phase which is the highest reported concentration, achieved by extractive fermentation [82]. However, one reaction product was supplemented in high concentrations and a high lipase concentration of 5 g L^−1^ was used. Co-cultivation could be useful to produce both butyrate and butanol. For this purpose, Cui et al. cultivated *Clostridium beijerinckii* and *C. tyrobutyricum* in a co-culture [14]. Butanol production of *C. beijerinckii* started first and butanol was present in a concentration 6.9 g L^−1^ when the growth of *C. tyrobutyricum* started. The authors reported that the butyrate production through *C. tyrobutyricum* in the co-culture was 39.0% less compared to the mono-culture and explained this with the solvent toxicity of butanol [14]. The esterification step was conducted after the fermentation in the same vessel by adding the hexadecane phase and lipase. A butyl-butyrate concentration of 5.1 g L^−1^ was measured. Carrying out a simultaneous co-cultivation and esterification could presumably prevent the product inhibition of the solvents and, therefore, increase butyrate and ester production. The ester product spectrum can be expanded by combining further strains. For example, *C. acetobutylicum* and *Actinobacillus succinogenes* were co-cultivated to produce 2.2 g L^−1^ of butyl-acetate [44]. However, the amount of enzyme used, which was 20 g L^−1^ is almost ten times the amount of product formed. To achieve an economically viable process, the amount of enzyme must, therefore, be significantly reduced.

A limiting factor of all applications is the product concentration that can be provided by the fermentation step. These concentrations are often too low to achieve high ester concentrations regarding the reaction equilibrium of the esterification and the extraction. Moreover, a pH below 4.8 (pKa butyric acid) is favoured for the esterification step as only undissociated acid can be converted to ester. In contrast, for acid production with clostridia, a pH around 6 is optimal [82]. The compromise in pH could, therefore, be a reason for low ester yields. In view of the high concentrations of enzymes used, the use of immobilised enzymes and the re-use of enzymes should also be considered. There are also investigations to utilise metabolic engineering to introduce the gene to synthesise the ester into the fermenting microorganism. A common method is to use the ATF1-encoded yeast alcohol acetyl transferase from *S. cerevisiae*. There are promising results with the metabolic engineering of clostridia strains and *E. coli*, where ester concentrations of up to 20 g L^−1^ could be achieved [20, 63]. Cells could also be used in an immobilised form or recycled by cell retention. It is also possible to carry out a surface display of lipase. Lu et al. demonstrated that a surface display of lipase on *E. coli* resulted in an esterification efficiency of 85% compared to the commercial enzyme [43]. A re-use of the immobilised cells with surface display was possible for five batches without loss of enzymatic activity. This could be a good alternative to the supplementation of free enzyme.

All in all, the combination of a fermentation and an enzymatic esterification shows potential for the bioproduction of ester. Zhang et al. showed that high concentrations of ester (17.3 g L^−1^) can be synthesised [82]. As the authors did not carry out the process in a separate variant, it is not possible to make a direct comparison in these cases. However, the simultaneous process variant could be especially interesting for ester synthesis, because the continuous degradation of fermentation products by esterification could open up the possibility of a continuous process. Simultaneous esterification could prevent fermentation products from accumulating, thus allowing continuous fermentation. By applying a feed flow and the continuous stripping of the organic phase the ester could then be produced in a continuous process. Though, the use of a continuous process only makes sense if it is possible to maintain the enzyme in the system. This could be achieved, for example, by enzyme immobilisation or a surface display of the enzyme. In general, the application of a continuous process has several advantages including an increase in productivity but also reduced downtime for cleaning and sterilization and the prevention of substrate and product inhibition [38]. This of particular interest to create an economically competitive process.

### Coupling fermentation and enzymatic hydrolysis in food industry

Another interesting application area of simultaneous fermentation and biotransformation is the production of lactose-free dairy products. Yoghurt is the best-known product of milk fermentation. Milk fermentation is typically carried out using mixed cultures of *Streptococcus thermophilus* and *Lactobacillus delbrueckii* subsp. *Bulgaricus* [50]. The mutual interaction of this bacterial consortium is responsible for the typical texture, flavour and acidic taste of yoghurt [5]. Within this fermentation lactose is partly degraded and lactic acid is produced as a main product. To achieve a higher degree of lactose conversion for products with a low lactose content, the enzyme β-galactosidase is used. The enzyme β-galactosidase is derived from fungi, bacteria or yeast, commercially from *Kluyveromyces fragilis*, *Kluyveromyces lactis*, *Aspergillus oryzae* and *Bacillus circulans* [4]*.* The enzymatic hydrolysis of lactose can take place prior to fermentation or simultaneous to fermentation, which is also known as co-hydrolysis. Many authors have, therefore, focussed on the influence of these process variants on the final product. Table 3 summarizes these variants with a focus on the differences in the degraded lactose content.

**Table 3:** Comparison of yoghurt production processes for low lactose content

Literature	Substrate	Process, conditions	Lactose conversion, β-galactosidase*	Final lactose content/g (100 g)^−1^	Lactic acid quanti-fication		Process time
[72]	Cow milk	Fermentation, 41 °C	35%	4.4	Titratable acidity, 1.1%		8 h
		Simultaneous, 41 °C	99%, *A. oryzae,* 0.9 U mL^−1^, 1 g L^−1^	0.1	Titratable acidity, 1.0%		8 h
[54]	Cow milk	Fermentation, 39 °C	12.6%	3.9	n.d		7 h
		Hydrolysis 4–6 °C, subsequent fermentation 39 °C	98.5%, *Bacillus licheniformis,* 18.1 U mL^−1^, 3.3 g L^−1^	0.3	n.d		4 h + 6.5 h
		Simultaneous 39 °C	84.2%, *B. licheniformis,* 17.3 U mL^−1^, 3.3 g L^−1^	0.7	n.d		6 h
[54]	Goat milk	Fermentation, 39 °C	14.0%	3.9	n.d		7 h
		Hydrolysis 4–6 °C, subsequent fermentation 39 °C	96.5%, *B. licheniformis,* 18.1 U mL^−1^, 3.3 g L^−1^	0.2	n.d		4 h + 6.5 h
		Simultaneous 39 °C	82.7%, *B. licheniformis,* 17.3 U mL^−1^, 3.3 g L^−1^	0.8	n.d		6 h
[31]	Camel milk	Fermentation, 42 °C	27.0%	3.3	Titratable acidity, 0.7%		7.5 h
		Hydrolysis 40 °C, 150 rpm, subsequent fermentation, 42 °C	90.0%, *K* *lactis,* 2.6 U mL^−1^, 0.5 g L^−1^	0.4	Titratable acidity, 0.8%		5 h + 4.8 h
		Simultaneous, 42 °C	71.2%, *K* *lactis,* 2.6 U mL^−1^, 0.5 g L^−1^	1.3	Titratable acidity, 0.8%		4.6 h
[77]	Bulk bovine milk	Fermentation, 42 °C	18.7%	3.8	HPLC, 6.9 mg g^−1^		3.9 h
		Simultaneous, 42 °C	91.4%, *K. lactis*, 0.4 g L^−1^, activity unknown,	0.4	HPLC, 6.5 mg g^−1^		4.3 h
[45]	Milk powder	Fermentation, 43 °C	19.8%,	4.6	n.d		3.9 h
		Simultaneous, 43 °C	97.9%, *K. lactis* and *A. niger*, 0.5 g L^−1^ activity unknown,	0.1	n.d		4.3 h

^*^Enzyme activity calculated from manufacturer information, enzyme activity related to the yoghurt volume

n.d.: not detected

The simultaneous process variant was already investigated by Toba et al. in 1986 and resulted in an almost complete conversion of lactose [72]. In contrast, the single fermentation with yoghurt starter cultures only achieved a conversion of 35.0%. Accordingly, the yoghurt which was enzymatically treated contained more glucose and galactose (3 g and 3.6 g, respectively), whereas the untreated yoghurt contained no glucose and only 1.2 g galactose in 100 g of yoghurt. To determine the acid content in food, the titratable acidity is a typical parameter. It determines the amount of a standard base (1 M NaOH) needed to adjust the pH of a specific volume or weight of sample to 8.2 (pH indicator phenolphthalein) [49]. For the fermentation of lactic acid bacteria, the result is then typically indicated in grams of lactic acid per 100 g of sample as lactic acid is the main product. As a result, the value is not as accurate as an HPLC (high-performance liquid chromatography) measurement, for example. Toba et al. measured the titratable acidity and determined a slightly lower acid content of 1% in the enzymatically treated yoghurt compared to 1.1% with a single fermentation [72]. Venica et al. measured the lactic acid concentration by HPLC and made a similar observation calculating 5% less of lactic acid with the simultaneous enzymatic lactose degradation compared to a single fermentation [77]. This was explained with an inhibition of the bacterias' lactose metabolism through high galactose and glucose concentrations resulting from the hydrolysis. However, Ibrahim et al. found a lower titratable acidity of 0.7% with the single fermentation [31]. A preceding enzymatic hydrolysis of lactose as well as the simultaneous hydrolysis resulted in a titratable acidity of 0.8%. These different results could be related to the use of different types and ratios of yoghurt starter cultures. In the production of lactose-free products, lactose conversion is the most important factor. Here it is particularly interesting to compare how the conversion of pre-hydrolysed milk differs from the simultaneous hydrolysis and fermentation. Popescu et al. achieved a nearly complete conversion of lactose if the hydrolysis was carried out before fermentation for cow and goat milk [54]. Within a simultaneous hydrolysis and fermentation of lactic acid bacteria the conversion was approximately 14% lower for both substrates. However, different temperature conditions were used for the separate enzymatic hydrolysis than for the simultaneous hydrolysis. Ibrahim et al. made the same observation for camel milk and found a conversion rate of 71.2% with the simultaneous process, which was almost 20% less than with prior enzymatic hydrolysis [31]. The exact reason for this inhibition during simultaneous hydrolysis is unclear. Products or metabolites of the fermentation could possibly lead to enzyme inhibition. Despite this, Martins et al. could achieve a nearly complete conversion of lactose with the simultaneous hydrolysis and fermentation [45]. The lactose conversion was optimised by investigating optimal lactose concentration, enzyme concentration and the time of enzyme supplementation. A lactose start concentration of 59.0 g L^−1^, which was adjusted by adding a specific amount of milk and whey powder, resulted in the highest conversion by adding the enzyme at the beginning of the fermentation. However, increasing the lactose concentration to 91.0 g L^−1^ also led to almost complete conversion. Conventional cow or goat milk has a lactose content of about 47 g L^−1^ which is why this is not a limiting factor [68]. The lactose conversion could be increased by increasing the enzyme concentration. However, a lower enzyme concentration of 0.5 g L^−1^ also led to a high lactose conversion of 97.9%, but as the enzyme activity is not specified, comparison is difficult. Almost all products of simultaneous enzymatic hydrolysis in Table 3 qualify as low-lactose products as they contain less than 1 g lactose per 100 g of yoghurt [36]. The low-lactose products can also compete with traditionally fermented products in terms of flavour and consistency [31, 54]. However, the limit value for lactose-free products of 0.1 g lactose per 100 g cannot be met by all products. Nevertheless, the advantage of simultaneous hydrolysis is that the process time can be reduced by up to 5 h compared to a separate hydrolysis and fermentation.

Within a similar procedure, Men et al. used enzymatic biotransformation and a subsequent fermentation with lactic acid bacteria to modify jujube juice into a low-calorie and probiotic functional beverage [46]. Jujube fruits naturally contain cyclic adenosine monophosphate, which is known to have positive health effects. On the other hand, it contains high-calorie sugar, such as sucrose, D-glucose and D-fructose. Glucose and fructose can be enzymatically converted to D-allulose, which has a very low calorific value but still tastes sweet. However, the enzymatic conversion rate of glucose and fructose were only 13% and 18%, respectively [46].

Overall, the combination of a fermentation and enzymatic biotransformation in the segment of food industry can lead to a significant improvement in the nutritional value of food. The combined process enables the production of food with low lactose or low calorie content. Saving process time through the simultaneous process could also lead to a reduction in process costs, making the processed food more competitive in price.

## Potential future applications for simultaneous fermentation and biotransformation

One-pot processes offer great potential but have only been used in few cases so far. However, there are other processes for which this approach would be promising. The production of semisynthetic β-lactam antibiotics is one example of a potential one-pot process. β-lactam antibiotics represent one of the most relevant drug classes of antibacterial agents worldwide [39]. Here, the fermentation of penicillin or cephalosporin is coupled with the enzymatic hydrolysis. This synthesises the intermediates 7-aminocephalosporanic acid (7-ACA) or 7-aminodesacetoxycephalosporanic acid (7-ADCA) and 6-aminopenicillanic acid (6-APA), which are the basic building blocks for semisynthetic cephalosporin and penicillin antibiotics. In a second enzymatic step, the antibiotic products are formed in an amidation reaction with different acyl donors [18]. Research showed that the one-pot operation of the enzymatic steps is possible and results in product yields up to 87% [9, 17, 32, 53]. The demand to integrate the enzymatic conversion into the fermentation process is high, because the purification of the fermentation broth is very complex. The purification procedure involves filtration, solvent extraction, decolorization, and back extraction with base solution, crystallization, washing and drying for penicillin [67]. Cephalosporin is being purified with an even more expensive chromatographic method, which requires several steps [6]. The elimination of this purification procedure between the process steps would be desirable. Concerning this problem, Giacobbe et al. suggested a simpler process, where penicillin G was separated in a single extraction step with an organic solvent and this crude solution was used for enzymatic hydrolysis [24]. This procedure could enable a simultaneous fermentation and enzymatic hydrolysis. However, experiments showed a significant decrease in enzyme activity when working with the crude penicillin solution, suggesting that other components extracted from the fermentation broth inhibit the enzyme reaction [24]. Shen et al. determined the possibility of the extraction of penicillin G in a three-phase system and which recovered more than 90% of penicillin G what produced 6-APA with a purity of 98% by enzymatic hydrolysis [67]. Here, filtration and ultrafiltration are utilised as a pretreatment of the fermentation broth and a back-extraction of penicillin G is necessary. At this point, the purification of the fermenting broth seems to be unavoidable. This contradicts the possibility to couple the two processes in one pot. However, current research is aimed at further simplifying the production of semisynthetic antibiotics and optimising the enzymatic conversion, which could increase the interest in a coupled process variant.

Another process that combines fermentation and enzymatic catalysis is the production of the polylactic acid (PLA). PLA is currently attracting attention for its potential as a biopolymer, offering interesting properties such as biocompatibility, recyclability, non-toxicity and compostability [40]. This makes it interesting as an alternative to petroleum-based plastic. The production of PLA is based on the fermentation of lactic acid. Commonly used microorganisms for lactic acid production are Lactobacillus and Bacillus strains, which provide high lactic acid yields and productivities, and high acid tolerance [30]. Lactic acid can then be enzymatically polymerised either by ring-opening polymerisation of lactides or by direct polycondensation of lactic acid with lipase [25]. The direct polycondensation of lactic acid yields in lower molecular weight polymers, which is why ring-opening polymerization is the currently preferred method. However, this technique requires an intermediate step, where lactic acid is converted into lactide via dehydration and depolymerization, which takes place at temperatures over 200 °C [15]. The direct polycondensation by lipase would, therefore, be the simpler option regarding the possibility of a combined process. The possibility to apply an in-situ extraction of lactic acid to the fermentation is part of current research and shows potential using different organic solvents. Teke et al. use a mixture of tributyl phosphate, tri-n-octylamine and oleyl alcohol as extractant, while Gao et al. use tri-n-decylamine and oleyl alcohol [22, 71]. Both authors found an increase in the total lactic acid concentration of 62% and 68% and an increase in yield of 36% and 27%, respectively, with the in-situ extraction alcohol. To enable a one-pot process, the enzymatic reaction should take place in this organic phase, what could lead to undesirable side reactions regarding the alcohol. Enzymatic polymerisation often uses toluene as solvent [25]. Chuensangjun et al. carried out the lipase-catalysed polymerisation of lactic acid in toluene at 50 °C under a nitrogen atmosphere and obtained PLA [12]. There is also a Japanese patent stating that the extraction of lactic acid with toluene is possible at temperatures ranging from 60 °C upwards [73]. A coupled one-pot process could, therefore, be conceivable if the fermentation conditions can be adapted, although this has not yet been tested. However, the formation of a third viscous PLA phase could complicate this approach [12].

These examples shown above highlight the limitations of carrying out a fermentation and a biotransformation simultaneously. Understanding and determining exactly how these processes interact and inhibit each other can be challenging. In addition, finding the optimum process conditions for the simultaneous process is difficult. Modelling and simulation, in conjunction with model-based experimental design, emerge as valuable tools to address these complexities and determine optimal process conditions. However, the integration of artificial intelligence (AI) driven methodologies, particularly within the framework of pre-process intensification 4.0 (PI4.0), offers a transformative approach to overcome these challenges. PI4.0, an evolution of the industry 4.0 concept in 2011, leverages data-driven algorithms to model process intensification methods, predict potential problems, identify equipment required, and optimise process approaches [42]. For example, Sebayang et al. use an AI-driven model to optimize enzymatic hydrolysis and fermentation to produce bioethanol [65]. The model is used to determine the optimal operating parameters, including substrate loading, enzyme concentration, yeast concentration, temperature and agitation speed. This resulted in an optimized enzymatic conversion and ethanol concentration but also reduced process time and costs. In addition, there are approaches to model the simultaneous hydrolysis and fermentation that can efficiently predict the process course with low deviation to the experimental data [21]. Furthermore, AI-driven models facilitate process optimisation through real-time monitoring and adaptive control strategies, enabling dynamic adjustments to process parameters to maximise the desired outcomes while minimising resource consumption and waste generation [26]. Recently, the trends due to digitalisation have increased with the use of more AI generated tools to predict, plan, and optimise processes. Pointed out within this review, integrating multiple processes into a single process can lead to difficulties which are hard to predict. However, AI technologies, with their capacity for learning and adapting used data from different sources, simplify process intensification by identifying patterns to stabilise and optimise the system.

Already AI-driven modelling finds extensive applications in various fields of biotechnology for example in protein engineering. Moreover, design within AI-models can be used for structural modelling and predictions on enzymes regarding their efficiency, activity, and stability. In drug discovery and pharmaceutical use similar systems are used to discover new targets and predict their pharmaceutical value [58]. Furthermore, AI technologies help in working with big data as in the field of genome and metagenome analysis. Recent years have witnessed an increase in publications especially in the field of process intensification and machine learning and process engineering or chemical engineering with a total of 929 publications in 2020 [42]. In example, process intensification can be combined with the industry 4.0 model to process intensification 4.0, i.e., used from the Li et al. lab by a cloud based lab and reducing 70% of experimental time by using AI predictions which shows the efficiency in time reducing methods using AI technology [37]. In conclusion, AI-driven modelling represents an advancement in biotechnological process intensification and offers a toolkit for understanding, predicting, and optimising complex biotechnological processes.

## Conclusions

**Fig. 5:**
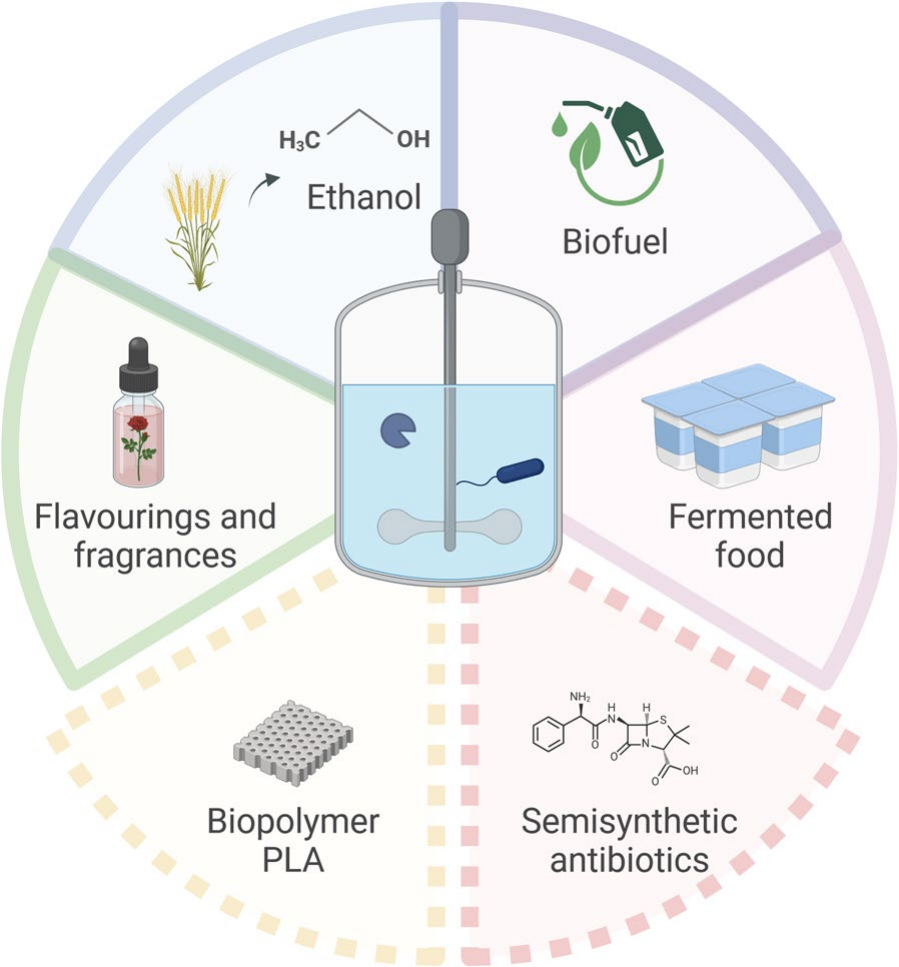
Overview of the fields of application of coupled fermentation and enzymatic biocatalysis

One-pot fermentation and enzymatic biocatalysis is an interesting process option that is used in a wide range of applications (see Fig. 5). In particular, these include processes aimed at more sustainable production solutions.

For all the processes discussed here, several advantages have been identified by combining fermentation and enzymatic biocatalysis in a single reaction vessel. These include the reduction of process time and vessels. This also results in a higher productivity, what could increase the economic efficiency of the process. The elimination of a separation step between the individual processes also leads to a simplification of the overall process and a reduction in process costs. Moreover, product inhibitions resulting from the fermentation or the enzymatic biocatalysis can be prevented, because the reaction products are continuously processed into the end-product. Many processes also include in-situ product removal, which further increases process efficiency and could make continuous process models feasible. Nevertheless, the influence of the individual processes on each other is difficult to understand and process control is challenging. It, therefore, makes sense to first consider and optimise the individual steps to better understand the overall system. However, a simultaneous process is not possible for all combined processes. When the optimal process conditions for the separated processes differ greatly, a compromise has to be found, which does not work for all processes. Then, temperature or pH shifts are a possible tool to solve this problem. The use of artificial intelligence in process planning and modelling could be an important tool in the future to predict the optimal process conditions. Furthermore, by-products of the fermentation could also lead to an inhibition of enzyme or to undesirable by-products. For these processes, a combination of ermentationn and enzymatic biocatalysis in one pot is not an option. Especially for products with high purification requirements, such as pharmaceuticals, it is necessary to maintain product separation between process steps. However, the possibility to combine the processes should always be considered to reduce process costs. Particularly, in the biotechnological production of sustainable products, it is important to achieve low process costs to compete with alternative processes based on fossil resources. The combination of fermentation and enzymatic biocatalysis could, therefore, be an interesting option to help move towards more sustainable production processes.

## Abbreviations

SHF: Separated hydrolysis and fermentation
SSF: Simultaneous saccharification and fermentation
Aqu.: Aqueous phase
Org.: Organic phase
HPLC: High-performance liquid chromatography
7-ACA: 7-Aminocephalosporanic acid
7-ADCA: 7-Aminodesacetoxycephalosporanic acid
6-APA: 6-Aminopenicillanic acid
PLA: Polylactic acid
AI: Artificial intelligence
